# Investigating the effectiveness of a HyFlex cyber security training in a developing country: A case study

**DOI:** 10.1007/s10639-022-11038-z

**Published:** 2022-04-11

**Authors:** Livinus Obiora Nweke, Anthony Jnr Bokolo, Gibson Mba, Emeka Nwigwe

**Affiliations:** 1 Norwegian University of Science and Technology, Trondheim, Norway; 2 Noroff Accelerate , Oslo, Norway; 3 Department of Applied Data Science, Institute for Energy Technology (IFE), Halden, Norway; 4 Foretrust Digital Consulting, Enugu, Nigeria; 5 Assiniboine Credit Union, Winnipeg, Canada

**Keywords:** IT in education, Blended learning, HyFlex pedagogy, Digital learning, Cyber security training, Developing country

## Abstract

HyFlex termed as hybrid-flexibility is a teaching approach where teachers and students have the alternative to participate in planned courses either remotely or face-to-face. This study examines the effectiveness of the HyFlex pedagogical method to teach highly interactive digital and face-to-face cyber security training in Nigeria amidst the pandemic. Data was collected using a survey questionnaire from 113 participants to evaluate student’s perception towards the effectiveness of the Hyflex method using physical and Zoom teleconferencing which allow students to participate remotely in the cyber security training. The developed questionnaire comprising both open-ended and Likert-style questions was administered to purposely sampled participants. Findings from this study presents implementation details on how the HyFlex teaching model was implemented from a developing country context. Besides, findings present challenges and opportunities experienced with adopting the HyFlex pedagogical model, and also offers recommendations to other instructors for employing this teaching model. Findings also reveal that although there were challenges experienced by the students who attended via online such as connectivity issues, competency in using some features in Zoom-conferencing, etc. The students did appreciate the flexibility HyFlex teaching afforded, indicating that HyFlex is a promising teaching approach for fostering engagement of students especially in large-group cyber security courses.

## Introduction

With digital learning becoming increasingly adopted in educational institutions, innovative approaches for deploying digital technology to promote course curricula development and implementation have emerged over the years (Binnewies & Wang, 2019). However, evidence from Heilporn and Lakhal (2021) revealed that online based learning is faced with some drawbacks, such as high dropout of students as learners often feel isolated. This could be linked to limited support provided by the instructor resulting in decreased student learning satisfaction as compared to face-to-face learning. Findings from the literature argued that both face-to-face learning and online based learning such as synchronous and asynchronous learning, hybrid learning, flipped classroom, blended learning, etc. are now widely employed in higher education (Bokolo, 2021; Foust & Ruzybayev, 2021).

In comparison, approaches such as blended learning employ synchronous student-instructor and student–student learning which yield improved student satisfaction as compared to traditional face-to-face or only online learning (Heilporn & Lakhal, 2021). While blended courses entail the combination of face-to-face and online teaching and learning activities (Bokolo et al., 2019), new blended approaches have also developed over the last decade to achieve cost-optimization and strengthen the accessibility and flexibility of students learning in educational institutions. One of these new blended approaches is labeled as multi-access, synchromodal, or HyFlex courses which mainly substitute physical sessions with digital synchronous learning for some or all students (Foust & Ruzybayev, 2021; Miller et al., 2021). The HyFlex pedagogical method employs digital asynchronous learning with flexible-synchronous learning where students opt to attend face-to-face (F2F), digital synchronously, or digital asynchronously via videos and recordings towards achieving flexible participation (Heilporn & Lakhal, 2021).

The HyFlex approach was first proposed by Beatty in 2006 (Beatty, 2007), as a medium to address physical space limitations and to well accommodate all students (with different background, knowledge and/or plan) (Foust & Ruzybayev, 2021). Specifically, Hyflex is described as multimodal learning that aims to create a highly flexible approach that assists students in accomplishing better course outcomes (Zehler et al., 2021). Presently, in the sphere of blended learning and online learning there is a lack of literature focusing on the adoption of Hyflex teaching approach as a modality in training cyber security professionals, amidst the pandemic which has led to more demand for Hyflex pedagogy in higher education. In this respect, there is need for studies that contributes to the limited literature on HyFlex as research that provides practical and theoretical evidence on blended learning and online learning methods utilizing both digital synchronous and asynchronous learning to improve students learning are not effectively implemented (Binnewies & Wang, 2019).

Prior studies have investigated students’ perceptions towards the adoption of blended, hybrid, and flipped learning (Kohnke & Moorhouse, 2021). However, there are fewer studies that explore students’ perceptions of HyFlex. This is because the HyFlex approach is new and is rarely implemented as a feasible alternative to online or face-to-face modes of learning and teaching. It is, therefore, important to assess learners’ perceptions of the HyFlex teaching approach from a developing country context. Furthermore, there is a need for cyber security professionals across the world due to digitalisation and high risk of security and privacy breaches. As of 2020 in countries such as the United States, there are nearly a third of cyber security vacant positions (Straub, 2021). The situation is further exacerbated in developing countries where there are insufficient resources to attract cyber security talents. This has prompted the World Bank to recently announce the launch of a new ‘Trust Fund on Cybersecurity’ (World Bank, 2021). Hence, there is an urgent need for professionals with skills and competence in cyber security around the world, especially in developing countries.

Therefore, this current study aims to examine the following research questions:

- How can HyFlex be implemented to utilize digital technologies in a cyber security training?
- What factors influence students’ perception on use of digital synchronous and asynchronous learning in a cyber security training?

Accordingly, this study provides evidence on the effectiveness of the HyFlex pedagogical method to teach cyber security training amidst the pandemic in a developing country context. This study reports on the transformation of an operations course into a HyFlex modality to enhance student engagement through regular flexible-synchronous sessions as well as equivalent T&L activities across course sections. HyFlex courses are characterised by a mixture of online and face-to-face learning components. In particular, students are allowed to choose to complete any part of the course in online and/or face-to-face mode. This current study helps to fill this gap in knowledge by presenting the experiences of students enrolled in a cyber security training via HyFlex mode.

Findings from this study reveal the challenges and benefits associated with adopting HyFlex and the implication of adopting HyFlex as an alternative method of instruction in higher education in response to the pandemic. Likewise, this study projects the students’ perspectives on how teachers can optimize and improve the current digital tools and pedagogical approaches they employ in HyFlex teaching. The remainder of this article is structured as follows. Section 2 presents the literature review while Section 3 presents the method employed in this study. Section 4 discusses the results while Section 5 presents the discussion and implications. Lastly, Section 6 concludes this study.

## Literature review

This section presents a discussion on the need for cyber security training in developing countries and why a HyFlex learning method offers a promising approach to address the growing cyber security skills gap. Also, an overview of HyFlex pedagogy, its benefits, and challenges are presented. And lastly, a review of the works related to HyFlex pedagogy is provided.

### The need for cyber security training

The main goal of this subsection is to examine the need for cyber security training in developing countries. With the ongoing digital transformation and the current efforts towards a sustainable digital economy around the world, developing countries are also not left out in this rapid digitalisation. In fact, digitalisation technologies such as information and communication technologies have been recognised by the United Nations Sustainable Development Goals as enablers of sustainable development (Velden, 2018). This is because of the potential benefits that come with digitalisation and its potential ability to help developing countries to achieve peace and prosperity for their citizenry. Thus, several initiatives have been introduced by developing countries to take advantage of the tremendous opportunities provided by digitalisation.

Digitalisation involves the integration of digital technology in almost all areas of our modern life. It is one of the prerequisites for a digital economy and has the potential to change the way goods and services are rendered. Developing countries can obtain several benefits with digitalisation. For example, they can modernise legacy processes, accelerate efficient service delivery, and help lift many of their citizenry out of poverty. Also, by taking advantage of service automation and advanced processing, such as artificial intelligence and machine learning, developing countries can promote a digital culture to help the transformation of their entire country.

Although digitalisation holds so many promises for developing countries, it also raises several challenges with regards to protecting the confidentiality, integrity, and availability of computing devices and networks, hardware, and software, and most importantly, data and information (Nweke, 2017). One of these challenges is the rising cases of cyberattacks, which have tended to discourage the current drive towards digitalisation. In recent years, several developing countries have been victims of cyberattacks. For example, the national bank of Bangladesh fell victim to a cyberattack and was robbed (Gray, 2016). Similar cyberattacks were targeted at banks in Ecuador (Finkle & Valencia, 2016), the Philippines (Chi, 2016), and most recently, it was reported that Nigeria, South Africa, and Kenya recorded over two million phishing (cyber) attacks in the first half of 2021 (Punch, 2021). This scary outlook is further exacerbated by the shortage of the required cyber security skills and competency in these developing countries, which is another very important challenge of digitalisation faced by developing countries.

Consequently, cyber security training can be employed by developing countries as one of the strategies to address the shortage of the required cyber security skills and competency to minimise the risks associated with digitalisation. This would require the development of a flexible cyber security education with the potential of reaching a large number of participants to build cyber security capabilities to achieve greater cyber readiness. Accordingly, HyFlex learning method offers a flexible cyber security training delivery mode with the potential of reaching a large number of participants that can be adopted by developing countries to expand cyber security training to address the growing gap in the required cyber and digital security capability and capacity.

### Overview, benefits, and issues in HyFlex pedagogy

The HyFlex pedagogy has been defined by Beatty (2014) as the learning approach that “enables a flexible participation policy for students whereby students may choose to attend face-to-face synchronous class sessions or complete course learning activities online without physically attending class”. It is a variant of hybrid learning method, which is also referred to as a blended learning environment; where students have the additional flexibility to decide when and how they engage with the learning processes. The course content and materials are organised to meet the needs of students participating both in-person and online (Beatty, 2014). In addition, HyFlex pedagogy is usually designed in such a way that students have the necessary technological skills required to access the participation choices (Miller et al., 2013).

**Fig. 1:**
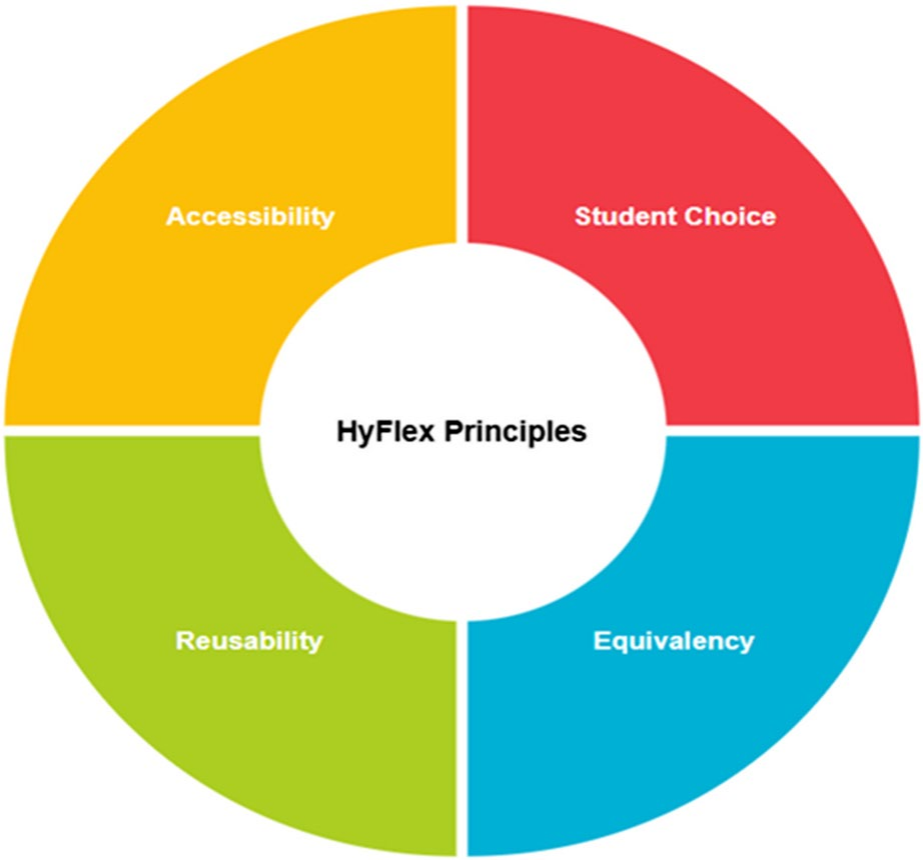
HyFlex principles

According to Beatty (2007), there are four main principles that can be applied when designing a HyFlex pedagogy and they include: student choice, equivalency, reusability, and accessibility. These principles, which are depicted in Fig. 1 are very important for the effective and efficient implementation of a HyFlex pedagogy. For example, student choice ensures that those interested in implementing a HyFlex pedagogy can “provide meaningful alternative participation modes and enable students to choose between participation modes weekly” (Beatty, 2014). The goal of this first principle is to ensure that HyFlex course design can provide students with the desired flexibility in how they complete the required course activities. To achieve this, those implementing HyFlex methodology need to have in mind that ensuring that students have the choice to decide how and when they engage with the learning processes is more important than imposing a particular learning style (Beatty, 2014).

The second principle of the HyFlex course design relates to equivalency. Equivalency ensures that those interested in implementing a HyFlex pedagogy “provides equivalent learning activities in all participation modes” (Beatty, 2014). This implies that regardless of the participation mode the student decides to engage with the learning processes, the learning outcome should be the same. As a result of this, the design of HyFlex course should ensure that students regardless of their participation mode, can be encouraged to reflect on their learning and to interact with their peers in a meaningful way that promote learning and the attainment of the desired learning outcomes.

Another principle of the HyFlex course design is referred to as reusability. Reusability aims to “utilize artifacts from learning activities in each participation mode as learning objects for all students” (Beatty, 2014). Several learning artifacts are usually obtained during both in-person learning activities and online learning activities. These learning artifacts for in-person learning activities may include video recordings, presentation files, handouts, etc., and those for online learning activities may include chats, asynchronous discussions, file posting, etc. The goal of reusability in the design of a HyFlex course is to ensure that students regardless of their participation mode can utilise these learning artifacts to support their learning process.

Accessibility as one of the principles of HyFlex course design ensures that students are equipped with technology skills and access to all participation modes (Beatty, 2014). HyFlex course design is based on the premise that students can decide in which participation mode they would employ to engage with the learning processes. This can only be achieved if the students have the prerequisite skills and access to technology needed for the implementation of the HyFlex course design. The implication of this is that those implementing HyFlex course design need to provide additional resources and training for both the students and the instructors to ensure that technological constraints do not limit the students’ choice of participation modes.

Nonetheless, it is important to note that a HyFlex pedagogy offers several benefits. For instance, students’ needs are taken into consideration by offering flexibility in course attendance (Abdelmalak, 2014; Miller et al., 2013). Also, it enables students to utilise similar technologies they use in their day-to-day activities for learning purposes (Thompson, 2013). Further, a HyFlex learning method enables those interested in implementing the learning approach to design course instructions to meet different students’ learning styles and preferences (Abdelmalak, 2014). Another benefit of HyFlex pedagogy is that it provides all students with equal opportunities to interact with other students and with teachers, regardless of the participation mode (Miller et al., 2013). And lastly, previous studies have revealed that HyFlex pedagogy promotes student engagement (Abdelmalak, 2014; Miller et al., 2013).

Despite the potential benefits of a HyFlex pedagogical method, there are also issues that need to be considered during its implementation. These issues, as has been observed by Binnewies and Wang (2019) are not only the ones peculiar to online instruction and face-to-face instruction, but also those of creating equivalency between the two modes to achieve the same learning outcomes. The implication of this is that the design and implementation of HyFlex pedagogy need to ensure students have the same opportunities for learning in either mode or that the learning outcomes are not negatively impacted because of choosing one mode over the other. Another important issue in the HyFlex learning method is student engagement. HyFlex pedagogy requires tremendous efforts to ensure student engagement because each participation mode would require unique student engagement methods to be effective (Binnewies & Wang, 2019).

In general, HyFlex pedagogy would provide developing countries with an alternative mode of delivering cyber security training to reach a large number of participants (Miller et al., 2013). This can be instrumental to building cyber security capabilities because a cyber security workforce has been identified as an important prerequisite to developing cyber readiness (Catota et al., 2019). In addition, HyFlex pedagogy can offer developing countries an opportunity to leverage their pool of cyber security experts around the world towards the training of cyber security professionals in their respective countries.

### Related works

The HyFlex learning format has been studied over the past few years. It was originally developed by Dr. Brain Beatty for his graduate course at San Francisco State University (Beatty, 2007). A survey of the literature as shown in Table 1 depicts a summary of prior studies on HyFlex adoption in higher education.

**Table 1:** Survey of prior studies on HyFlex adoption in higher education

Authors, year, and contribution	HyFlex approach	Methodology adopted	Context and Country
Kohnke and Moorhouse (2021) Adopted HyFlex in higher education in response to COVID-19 based on students’ perspective	Utilized face to face, Zoom video-conferencing application, other digital tools and mixed approach	Qualitative approach interview	-Students’ perspectives -Hong Kong
Vilhauer (2021) provided a background viewpoint on HyFlex and the deployment of a modified HyFlex model	Synchronous via Zoom breakout rooms and asynchronous with students using Padlet and Blackboard discussion boards and other online tools	Not reported	- Content, engagement, and assessment strategies for students -United States of America (USA)
Keiper et al. (2021) examined student perceptions on the usefulness of Flipgrid for HyFlex learning	Online video discussion board learning platform	Questionnaire	-Students’ perceptions -USA
Straub (2021) designed a HyFlex course for defensive security	HyFlex-based model was used which comprises of quizzes quizzes, experiential, lab exercises, and discussion boards	Not reported	-Cybersecurity education -USA
Foust and Ruzybayev (2021) explored students' academic experience with Hyflex teaching model	HyFlex instructional model	Survey	-Engineering courses -USA
Raman et al. (2021) provided practical guidelines for HyFlex undergraduate teaching amidst the pandemic	COVID-19 HyFlex model and Group Work	Not reported	- Facilitate effective peer collaboration in the classroom -USA
Brown and Tenbergen (2021) investigated teaching software quality assurance during COVID-19 based on the HyFlex approach	Lectures (physical class, with video recordings), face-to-face activities, group assignments, group projects, and exams via online campus management system	Quantitative evidence	-Efficacy of the HyFlex educational paradigm -USA
Lohmann et al. (2021) provided classroom management initiatives for Hyflex instruction	Best practices in Hyflex instruction for virtual and physical learning	Not reported	- Setting learners up for success in the hybrid learning environment -USA
Miller et al. (2021) presented pandemic teaching opportunities and challenges for teaching communication	HyFlex teaching approach (online and physical)	Not reported	-Improve teaching in HyFlex, BlendFlex, and remote courses -USA
Keshishi (2021) researched on playful reflective thinking within a HyFlex classroom	HyFlex teaching approach (online and physical)	Conceptual	-Students’ engagement -United Kingdom (UK)
Zehler et al. (2021) implemented a Hyflex simulation for creative method to unprecedented circumstances	Simulation and via online Zoom	Quasi-experimental study, Case study	-Support gains in critical thinking and judgment of learners -USA

Table 1 shows a review of survey of 11 studies that investigated HyFlex adoption in higher education. Besides, several other authors have also considered different aspects of the HyFlex learning method. For example, Kyei-Blankson and Godwyll (2010) explored the extent to which a student's needs and expectations are met in a HyFlex learning environment. They also compared instructor perspectives regarding participation and performance in HyFlex courses with previously face-to-face classes. Similarly, Abdelmalak and Parra (2016) considered student’s perspectives regarding HyFlex course design. They utilized qualitative study methods and focused on the participants' perspectives and experiences to obtain interesting results about HyFlex course design. The results obtained from the study indicated that HyFlex course design was able to accommodate student needs and their life circumstances, increase student access to course content and instruction, and gave students a sense of control over their learning.

The effectiveness of HyFlex courses has been examined by Lakhal et al. (2014). They employed two categories of variables one independent variable (course delivery mode) and four dependent variables (satisfaction, performance on multiple-choice test, written exam, and continuous assessment) and a total of 376 students participated in the study by responding to an online questionnaire. The findings from the study showed that no significant difference was found between students who choose different delivery modes on satisfaction, multi-choice test, and written scores; but significant differences were observed on continuous assessment scores. Miller et al. (2013) also evaluated the effectiveness of a HyFlex instructional model, specifically designed for large, on-campus courses. In this study, a total of 161 undergraduate students participated in the pilot section of a course while a control group that was made of 168 undergraduate students enrolled in two additional sections of the course. The results from the study revealed that the HyFlex instructional model had no negative impact on student performance in class, either in overall learning or individual grades and that the HyFlex model performed no differently from the traditional classroom model with respect to student learning.

Moreover, Binnewies and Wang (2019) explored equity and engagement methods to assist student learning in a HyFlex learning format. The approach they adopted in this study is to evaluate their teaching components according to student participation, and the quantitative and qualitative feedback received from the students. The results from the study observed that most students appreciated the HyFlex mode of delivery, however, it was constrained in some way by the technology available. Heilporn and Lakhal (2021) converted a graduated-level course into a HyFlex modality and then considered what are the effective engagement strategies. In this work, they combined both exploratory qualitative and mixed method approaches for data collection and analysis. The results obtained from this work demonstrated that HyFlex is a promising learning modality for encouraging student engagement at the graduate level, including large groups.

In contrast to all the works discussed in the preceding paragraphs, we investigate the effectiveness of deploying a Hyflex learning format for cyber security training in a developing country. With the growing demand for cyber security professionals around the world, especially in developing countries, there is a need for designing and delivering a flexible cyber security training that will accommodate participant’s needs and will provide greater access to cyber security training to a large number of participants. Thus, this study fills the gap in the existing literature by proposing a HyFlex cyber security training format as a promising training delivery mode that could expand cyber and digital security capability and capacity, especially in developing countries.

## Method

This section presents the environment, the methods and the data collection instruments used for the research.

### Research background

This study adopted a case study approach to investigate the efficacy (opportunities and challenges) of the Hyflex method in delivering cybersecurity training in a developing country. The environment (setting) of the study is Nigeria, a developing country where access to educational facilities, technology and supporting infrastructure is still a challenge compared to what is obtainable in advanced western societies. The candidates for the study were participants in a cyber security training delivered using a Hyflex method consisting of some participants receiving training via in-person, face-to-face and some joining virtually. The physical training venue was located in Southeastern Nigeria.

Also, the participants / learners were mostly graduates and students in tertiary institutions cutting across several regions of Nigeria. The instructors were subject matter experts in the field of cyber security with very good experiences in teaching using traditional and virtual methods. The instructors were located in three continents (Americas, Europe and Africa) while the participants were mostly resident in Nigeria with one participating from the USA. The training lasted for a period of twelve weeks starting from 07 August 2021 and ending on 23 October 2021.

In addition, the language of instruction was English. English language was chosen bearing in mind the background and location of the instructors and expected trainees. The students were evaluated by ensuring that they had at least 70 percent attendance on all training sessions and have completed all the training exercises and assignments including presentation of the badges from the two CISCO courses. This qualifies the participant for graduation and award of the programme’s certificate of participation.

### HyFlex method implemented

The HyFlex method was employed in this study in providing cyber security training for students in a developing country amidst the pandemic. The design of the HyFlex pedagogical method relied on the four main principles of the HyFlex course design proposed by Beatty (2007) and described in detail in Section 2.2. Using these four main principles of the HyFlex course design, the following subsections describe the design and development of the HyFlex method implemented in this study.

#### Student choice

Before the commencement of the training, the programme mode of delivery was explained to participants as HyFlex with each participant given a choice of deciding his/her preferred mode of participation (physical or virtual) with the option to combine or switch at will. The training classes were held weekly on Saturdays for three hours each day from 11.00 GMT to 13.00 GMT.

Furthermore, several digital technologies were employed for the training including Zoom Meeting link for online class participation, WhatsApp Instant messaging for instant messaging and group communication, Slack for messaging and group collaboration and Email for messaging and communication. These different digital technologies enabled the participants to choose their preferred mode of attendance according to their needs, circumstances and learning requirements.

#### Equivalency

The Zoom meeting platform used for the training provided students a measure of equivalency with the opportunity for students joining remotely to interact synchronously via chat tool and microphone and asynchronously using the Slack channel used for the learning activities. The Zoom meeting platform also allowed the instructors to share the course curriculum, practical demonstrations, and learning resources to all students regardless of their mode of participation (either joining remotely or in-person).

Additionally, a week before each training, the learning materials for the week were posted on Slack which every candidate was able to access and read before the training meeting day. Added to that was a set of discussion questions based on the reading materials which the candidates were expected to research and answer. The reports and answers were required to be posted to the Slack forum for the instructors and fellow learners to read and review. Another set of questions, known as Reflection questions, are equally posted which the candidates were expected to answer and forward their answers directly to the instructors for review and feedback. Furthermore, every candidate enrolled in the training was also expected to enroll in two CISCO training and certification programmes. To go through the two programmes on their own, complete it and obtain training badges as evidence of success.

#### Reusability

The concept of reusability in the design of the HyFlex pedagogical method as applied in this study relates to the training materials provided for all the students regardless of their mode of participation. All students had access to the training materials and additional learning artifacts obtained during in-person learning are shared as learning objects for all students. In the same way, learning activities completed by online students, such as chats, asynchronous discussions, file posting, etc., are also shared as learning objects for all students.

#### Accessibility

All the students enrolled into the training are equipped with the required technological skills and access to all participation modes. During the first synchronous meeting, we demonstrated to all students the use of the Slack channel, specifically navigation support. Before the training sessions begin, the Zoom link was posted in the Slack channel so that the students can easily join the training session. For students who due to lack of Internet access or other technical reasons cannot join the training sessions remotely, they were able to join in-person.

Another key aspect of accessibility that was integrated in the design of the HyFlex method implemented relates to how the course materials and learning activities were accessible to and usable for all students. The course materials used for the training were publicly accessible information. The other learning activities that took place via the Slack channel were also accessible to all students. This is because all students regardless of their participation modes are part of the Slack channel and were able to engage with the learning processes.

### Curriculum design

**Fig. 2:**
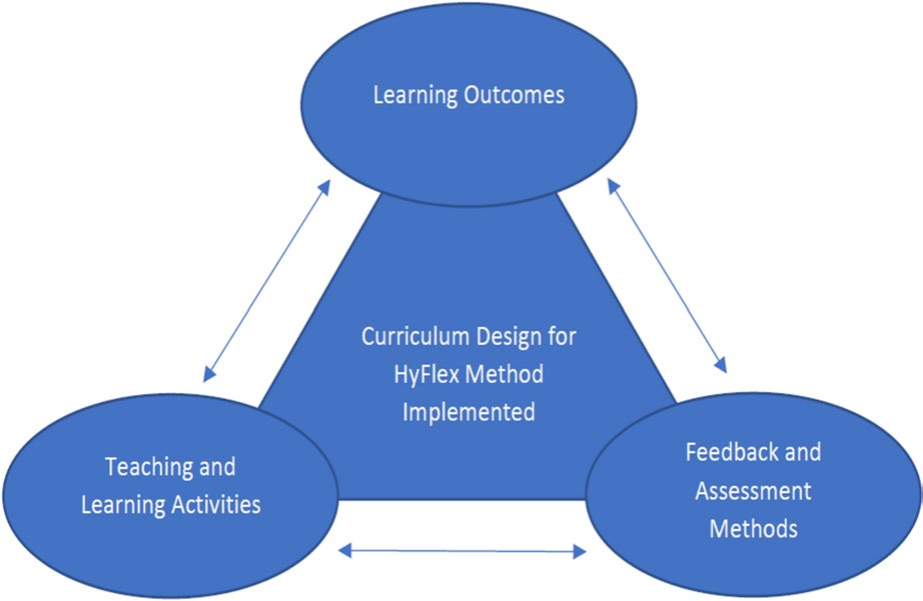
Curriculum design for HyFlex method implemented

The curriculum design employed for the HyFlex method implemented in this study was based on the principles of constructive alignment first developed by Biggs (2003). This is because constructive alignment framework is a widely accepted framework for curriculum design which aims to provide students with coherent, connected, and integrated learning experience. Constructive alignment as observed by Biggs (2003) has two aspects: “the 'constructive' aspect refers to the idea that students construct meaning through relevant learning activities”; and “the 'alignment' aspect refers to what the teacher does, which is to set up a learning environment that supports the learning activities appropriate to achieving the desired learning outcomes.” We utilised this approach to design the curriculum for the HyFlex method implemented in this study as depicted in Fig. 2.

Using the constructive alignment methodology as shown in Fig. 2, we first defined the learning outcomes for the implemented HyFlex cyber security training in terms of not only topic content, but also, the level of understanding we want participants to achieve. At the beginning of each topic, we clearly stated what we want the students to learn and how well the topic needs to be understood. In addition, the learning outcomes were also aligned with the feedback and assessment methods chosen for the training. This was to assist the students to develop the required knowledge as well as evaluate the extent to which they have acquired the required skillsets.

Similarly, we designed the teaching and learning activities to ensure that the defined learning outcomes were achieved and were supported using the feedback and assessment methods. The reading materials were shared with the students at the beginning of each learning week as well as discussion forum question, assignment and learning reflection to promote student learning and engagement before the training session. To stimulate discussions related to the topics introduced for the learning week, students were expected to response to the discussion forum question and the instructors were to coordinate and provide feedback to the ongoing discussions. During the training sessions, students were given the opportunity to ask for clarification about some of the concepts that are not clear from the topics presented during the learning week. Thus, the environment for the teaching and learning activities were setup to increase the likelihood that students will engage in the activities designed to attain the desired learning outcomes.

Lastly, we choose feedback and assessment methods that will assist in evaluating how well the students have achieved the defined learning outcomes. The feedback and assessments methods we employed for the training included the students’ response to the discussion forum questions, their submitted assignments, and their learning reflections. These methods were designed in such a way that students preparing for the assessments will be learning the curriculum. This was to ensure that the feedback and assessment methods were aligned to the learning outcomes of the implemented HyFlex cyber security training.

### Questionnaire design and data collection

The investigators used questionnaires for data collection and follow-up interviews to clarify unclear answers and to probe deeper into some responses that require follow up. Since all the learners are tech-savvy and have access to the Internet with most of them receiving instruction via remote access, an online questionnaire tool with automatic data aggregation and analysis capabilities (Google form) was used for data collection. This made for ease and convenience. The link for the questionnaire was forwarded to the training participants via WhatSapp, Email and Slack. The respondents were given two weeks to complete and submit the questionnaire forms with occasional reminders sent to them. The questionnaire was designed as a combination of multiple choice and open-ended questions with the questions framed to elicit the required answers that will enable us to answer the research questions. English language was employed in the design of the questionnaire. The questions were reviewed by an expert who confirmed they were appropriate for the study.

### Questionnaire validation

To validate the questionnaire and to ensure that it is error-free and enlisted the required type of answers/responses from the respondents, we administered it to five candidates selected at random. The responses received were consistent with our expectations, which confirms the adequacy of the questionnaire for the data collection for the research. The population size for the study consisted of the 113 learning candidates who registered for the training programme. Because of the limited size of the population, we decided to include all of them in the study eliminating the need for sampling. The sample size therefore consisted of 100% of the population. Regarding the sample size, a total of 113 candidates who participated in the training and who received the questionnaire, 47 questionnaires were returned representing 41.59% response rate which we considered adequate.

## Results

In this study the collected data was analyzed and reported using Microsoft Excel utilizing descriptive statistical analysis by employing percentage and frequencies to validate the objectivity and significance of the questionnaire items.

### HyFlex course participation

**Fig. 3:**
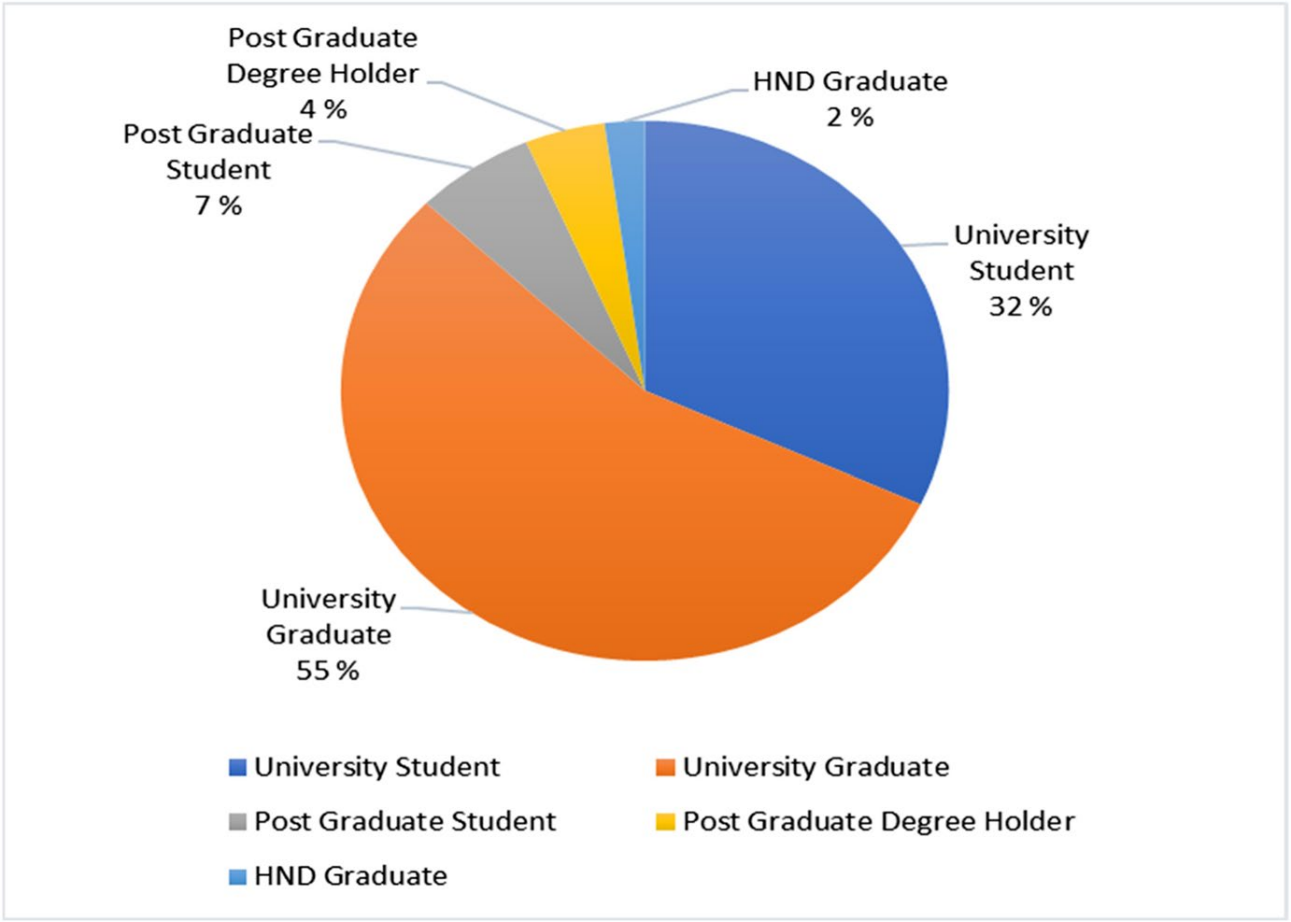
Distribution for respondents’ education level

Results from Fig. 3 suggest that the participants possess some educational qualifications. 55% (26) of the respondents are university graduates, 32% (15) are university students, and 7% (3) are postgraduate students. Also, 4% (2) are post graduate degree holders and 2% (1) are HND graduates.

**Fig. 4:**
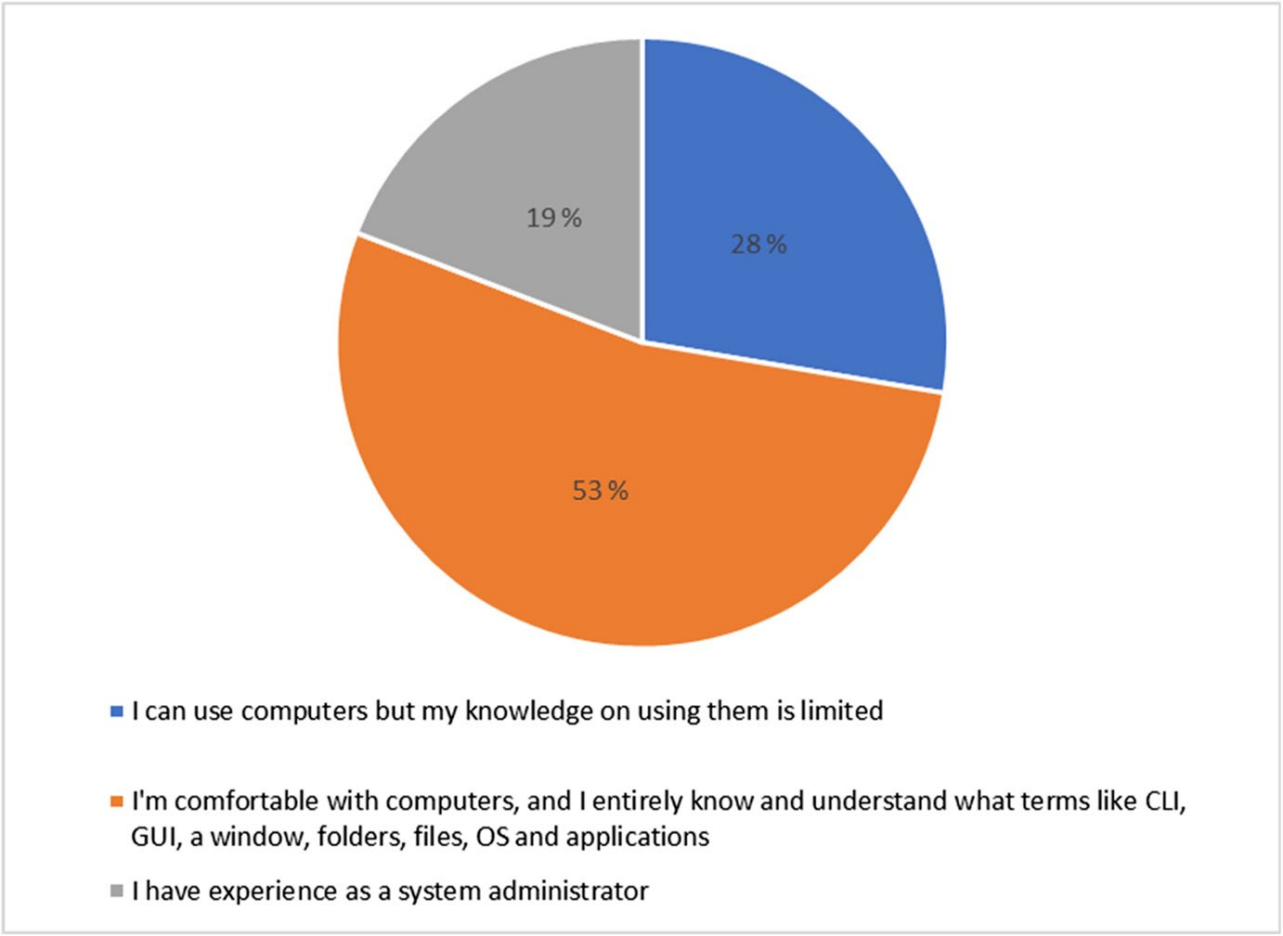
Distribution for respondents’ computer use level

Next, we assess the student’s level of using computers in general. Results from Fig. 4 indicates that 53% (25) of the students are comfortable with computers, and they have entirely knowledge and understanding of computer terms like command-line interface (CLI), graphical user interface (GUI), windows, folders, files, operating systems (OS) and applications. The results also show that 28% (13) of the students can use computers but their knowledge on using computers is however limited. Lastly, 19% (9) of the students have experience as a system administrator. This result suggests that all students are familiar and have knowledge on the use of computers for remote learning in the HyFlex approach.

**Fig. 5:**
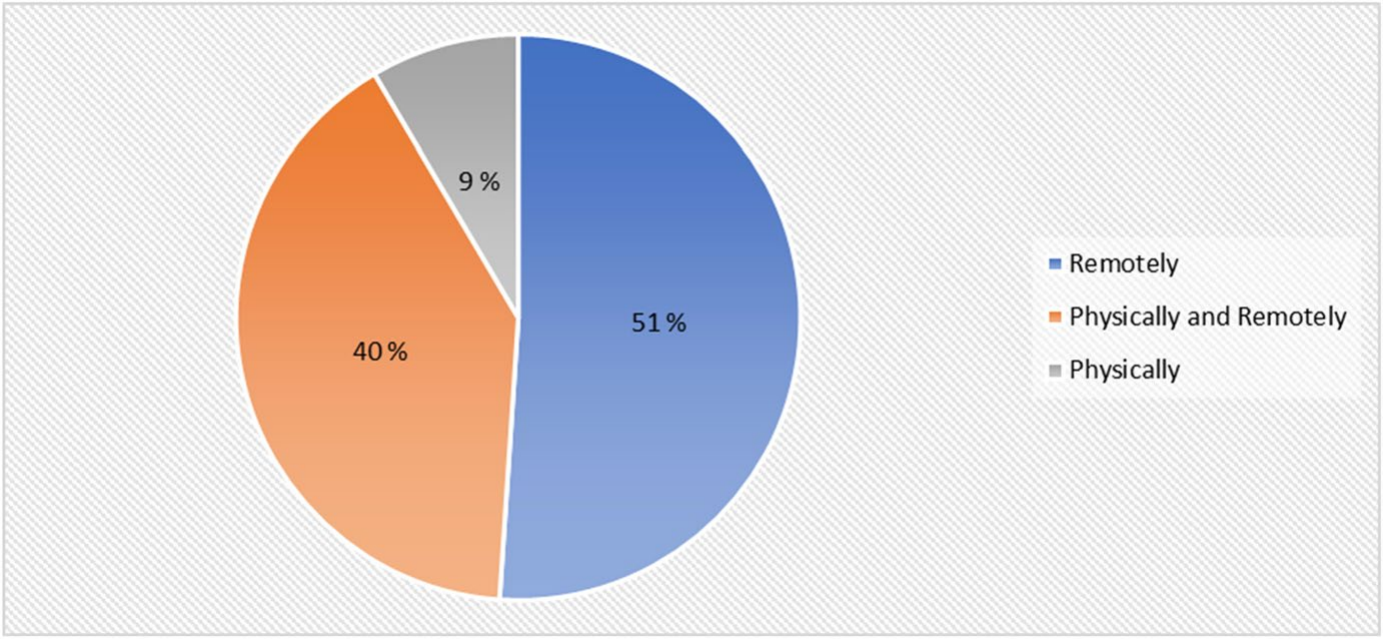
HyFlex mode preferred

Figure 5 presents results from the mode of HyFlex the students selected during the cyber security training. The result reveals that 51% (24) of the learners preferred and attended the remote training sessions, whereas 40% (19) of the students preferred blended learning which involved both face-to-face and digital learning, and lastly only 9% (4) of the students attended the physical class.

**Fig. 6:**
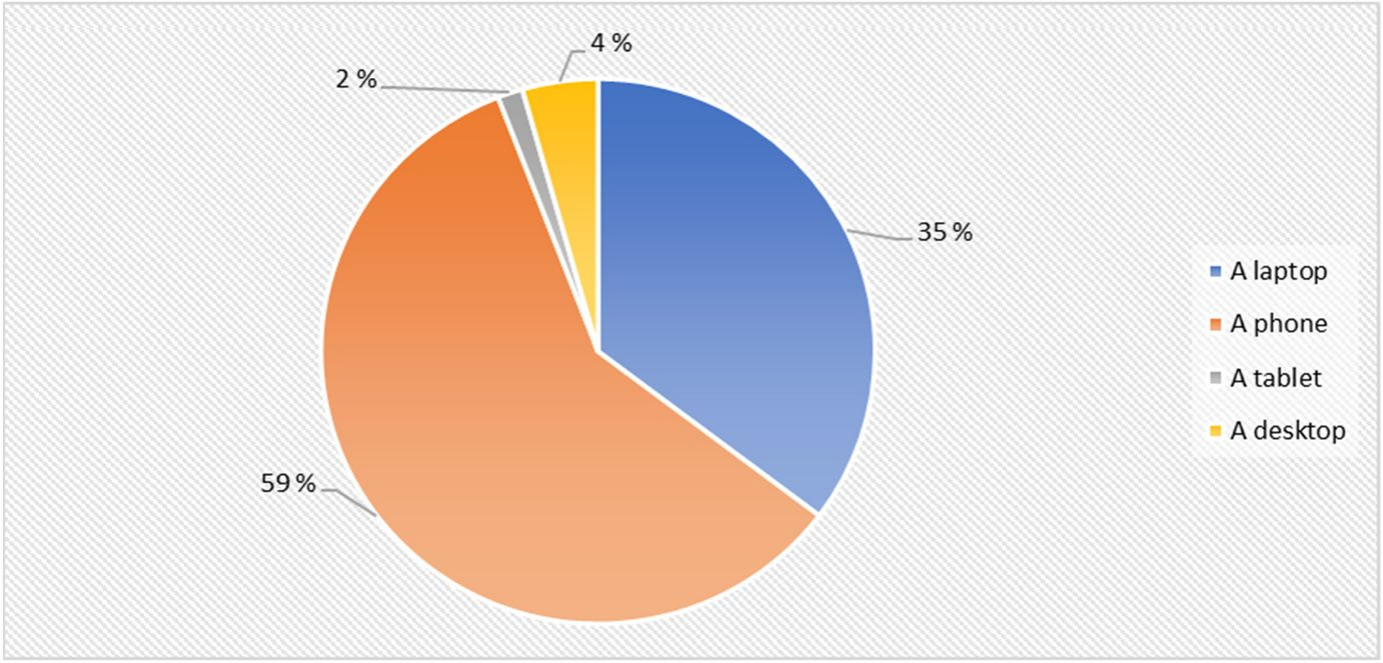
Distribution of devices used during the HyFlex cyber security training

Furthermore, the result as seen in Fig. 6 shows that as related to the device/devices used by the students to join the HyFlex cyber security training. Majority of the students utilized their mobile phones, and then other students used their laptops, desktop computer and lastly tablet devices for the digital lecture session.

The result in Fig. 6 suggests that mobile phones are well used in the developing country setting as many students use mobile learning as its affordable as compared to other devices employed in developed countries.

**Fig. 7:**
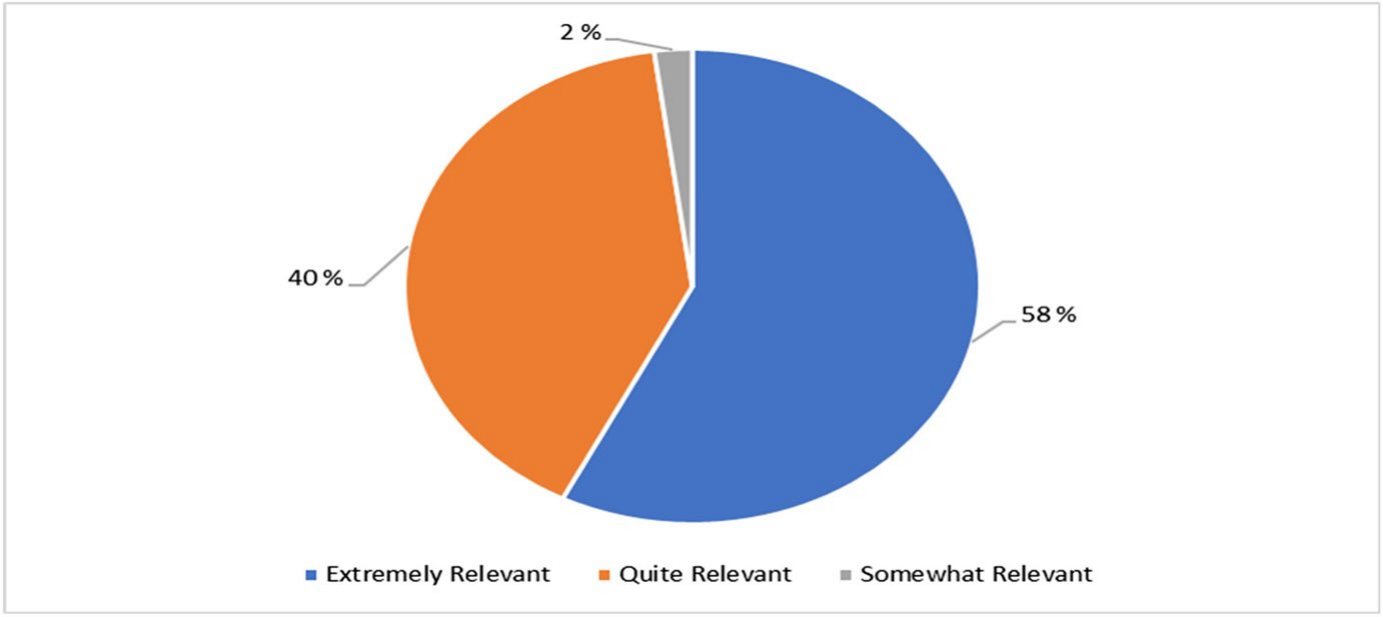
Relevance of the HyFlex cyber security training

Besides, the perception of the students towards the relevance of the cyber security training towards their career/school/overall life is measured as seen in Fig. 7. The result suggests that 58% (27) of the learners believed that the HyFlex lecture was extremely relevant and 40% (19) of the learners perceived that the HyFlex lecture was quite relevant. Finally, the remaining 2% (1) of the students believes that the training was overall somewhat relevant.

### Factors that impact students’ participation in the HyFlex training

**Fig. 8:**
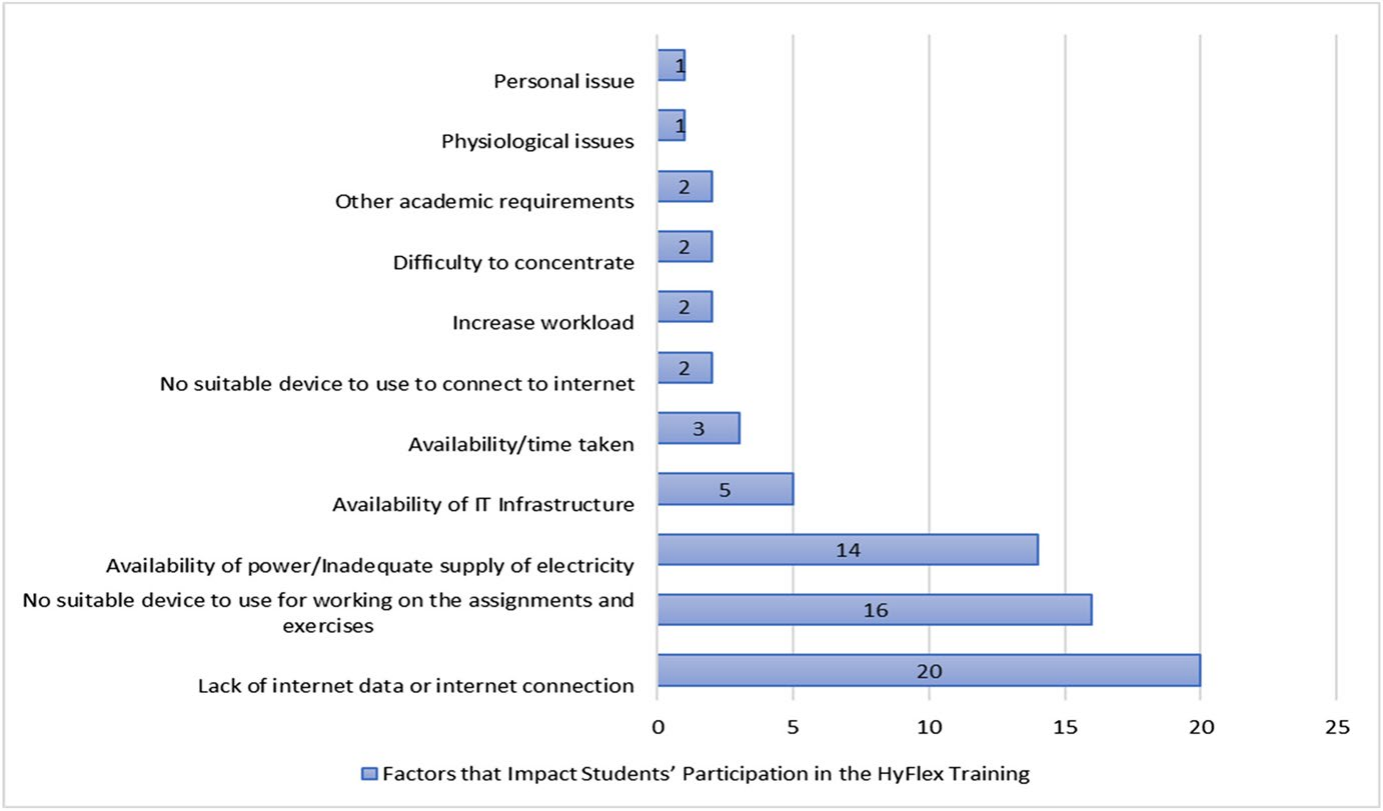
Factors that impact students’ participation in the HyFlex training

The student’s perception towards the factors that impact their ability to properly participate in the HyFlex training are presented in Fig. 8.

The results suggest that 20 students reported that there was a lack of internet data or internet connection as the need to purchase internet subscription to get access to the internet. Also, 16 students reported that there was no suitable device made available for the students to help with drafting and carrying out their assignments and exercises. Furthermore, 14 students were mostly affected by the availability of constant power supply/inadequate supply of electricity during the digital session of scheduled lectures.

The results also indicated that 5 students reported that the availability of IT infrastructure was one of the issues faced during the HyFlex cyber security training, 3 students were faced with other academic tasks that required their time. The result also revealed that 2 students were individually impacted by issues such as no suitable device to use to connect to the internet, increased workload, difficulty to concentrate and other academic requirements faced during the HyFlex training. Finally, 1 student reported being impacted by physiological and personal issues respectively as seen in Fig. 8.

### HyFlex course content and material

**Fig. 9:**
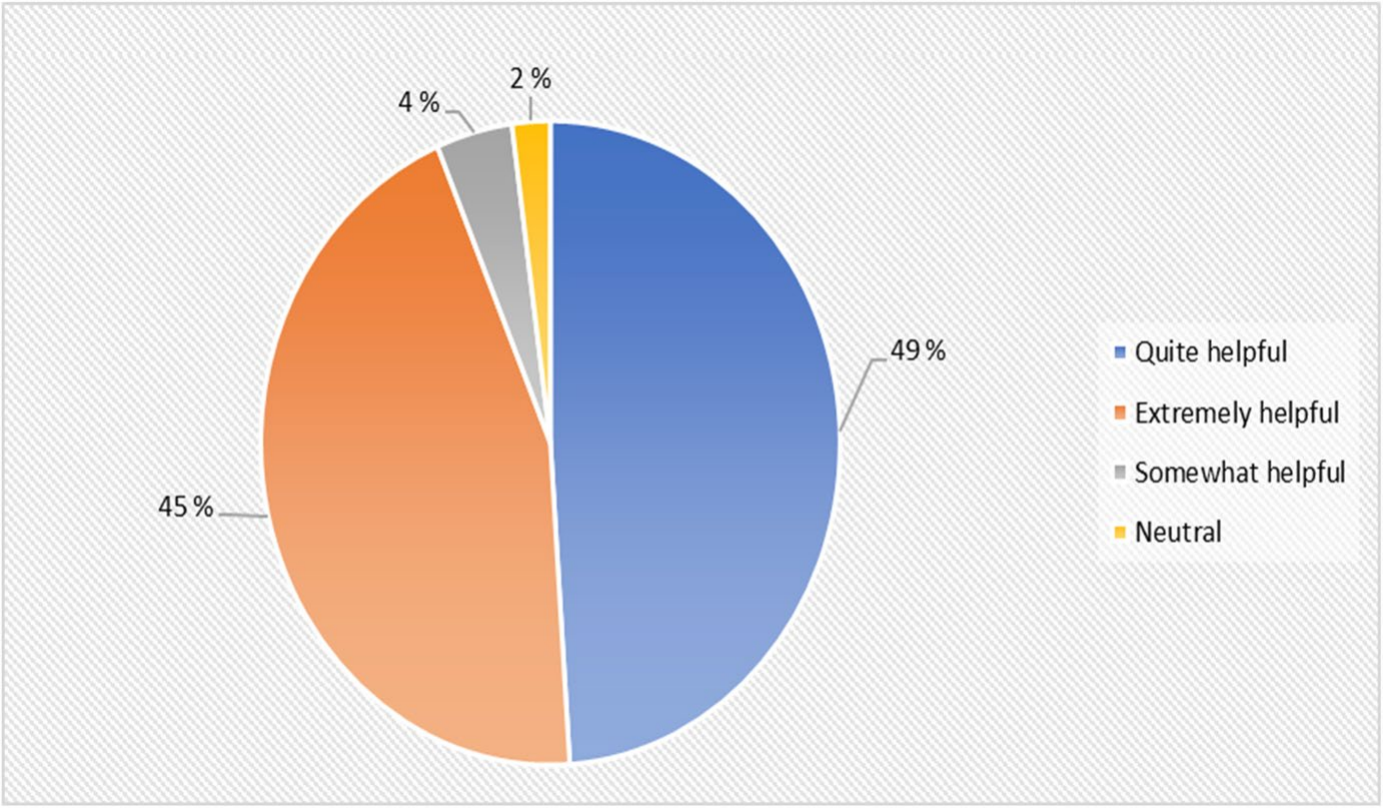
Usefulness of Hyflex course resources and materials provided

The students also provided their perception on how helpful the course resources and materials provided by the trainers on different cyber security topics covered. The results from their perception on course resources is shown in Fig. 9.

Results from Fig. 9 reveal that 49% (23) of the student perceives that the provided course materials from both online and physical lecture were somewhat helpful and 45% (21) agrees that the course materials were extremely helpful, 4% (2) stated that the course materials were quite helpful, and lastly 1% (2) mentioned that they are neutral regarding the course materials provided by the trainers.

### Facilitator's presentation approach

**Fig. 10:**
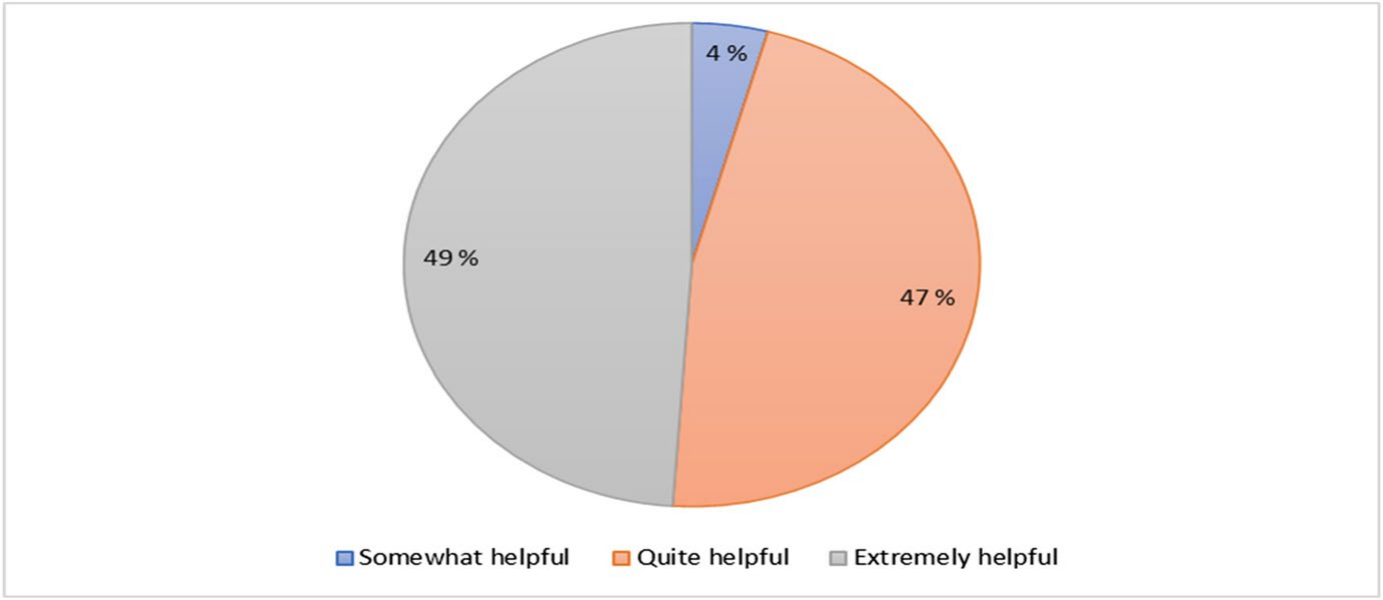
Distribution for respondents’ opinion on helpfulness of coordinators

Figure 10 shows the percentage distribution for respondents’ opinion on the helpfulness of coordinators during both physical and digital lectures. The result states that 49% (23) of the students perceive that the trainers from both online and physical lectures were extremely helpful and 45% (22) agree that the trainers were quite helpful, and finally 4% (2) stated that the trainers were somewhat helpful during the cyber security training.

**Fig. 11:**
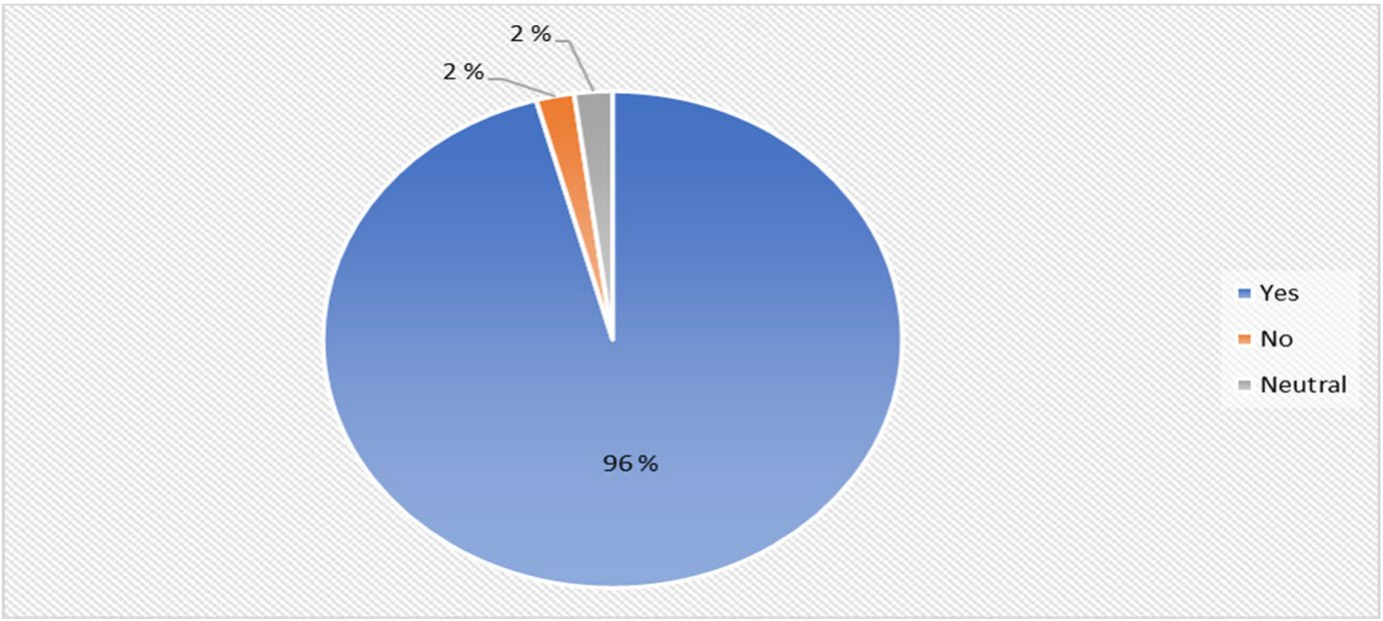
Experience of trainers to deliver cyber security training distribution

In addition, results from Fig. 11 suggest that the majority of the students believe that the trainers have knowledge on cyber security. The result stated that 96% (45) of the students indicated that the coordinators had requisite training and experience to deliver the program on cyber security training.

Furthermore, the questionnaire assessed how helpful were the coordinators in providing the students with support they needed during the HyFlex cyber security training.

**Fig. 12:**
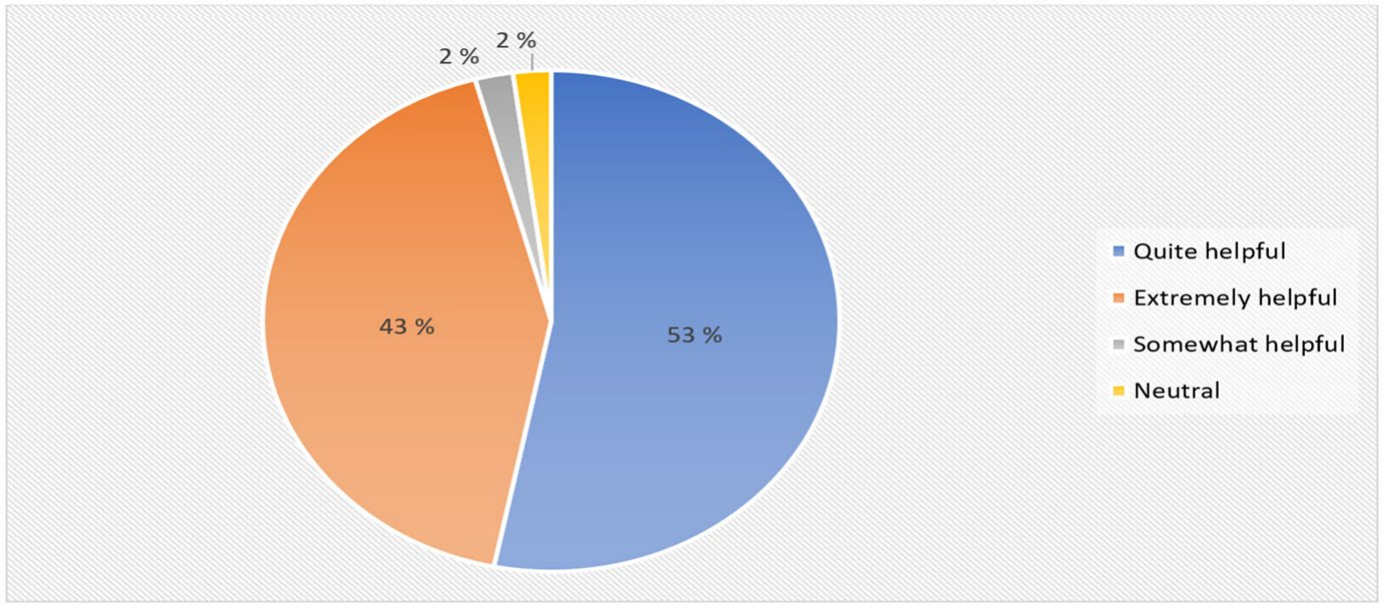
Helpfulness of the coordinators to the student’s distribution

The result as seen in Fig. 12 suggest that 53% (25) of the student perceives that the support provided by the trainers both during the online and physical lecture to the students were quite helpful and 43% (20) agrees that the provided support was extremely helpful, and 2% (1) stated that the support offered by the trainers were quite helpful and neutral respectively.

### Recommendation of the HyFlex course

**Fig. 13:**
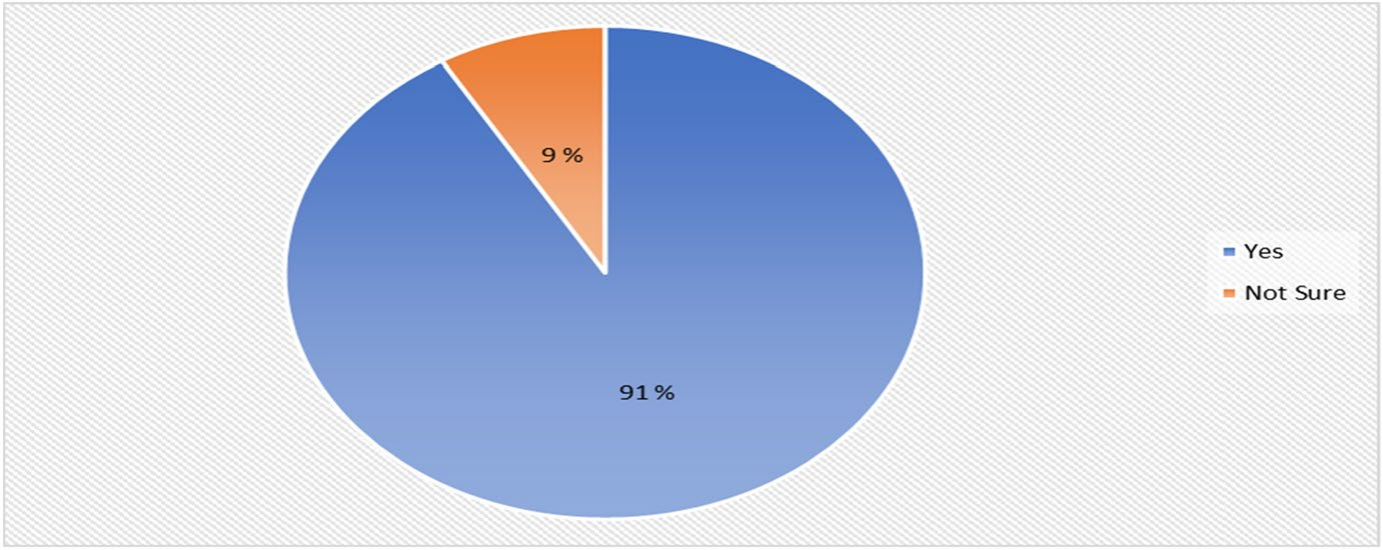
Enjoyment of the HyFlex course distribution

Figure 13 shows that many of the students 90% (43) of the students agreed that they were happy with the implemented HyFlex cyber security course, although 90% (4) students answered that they were not sure about the course content if it is acceptable or if it can be improved.

**Fig. 14:**
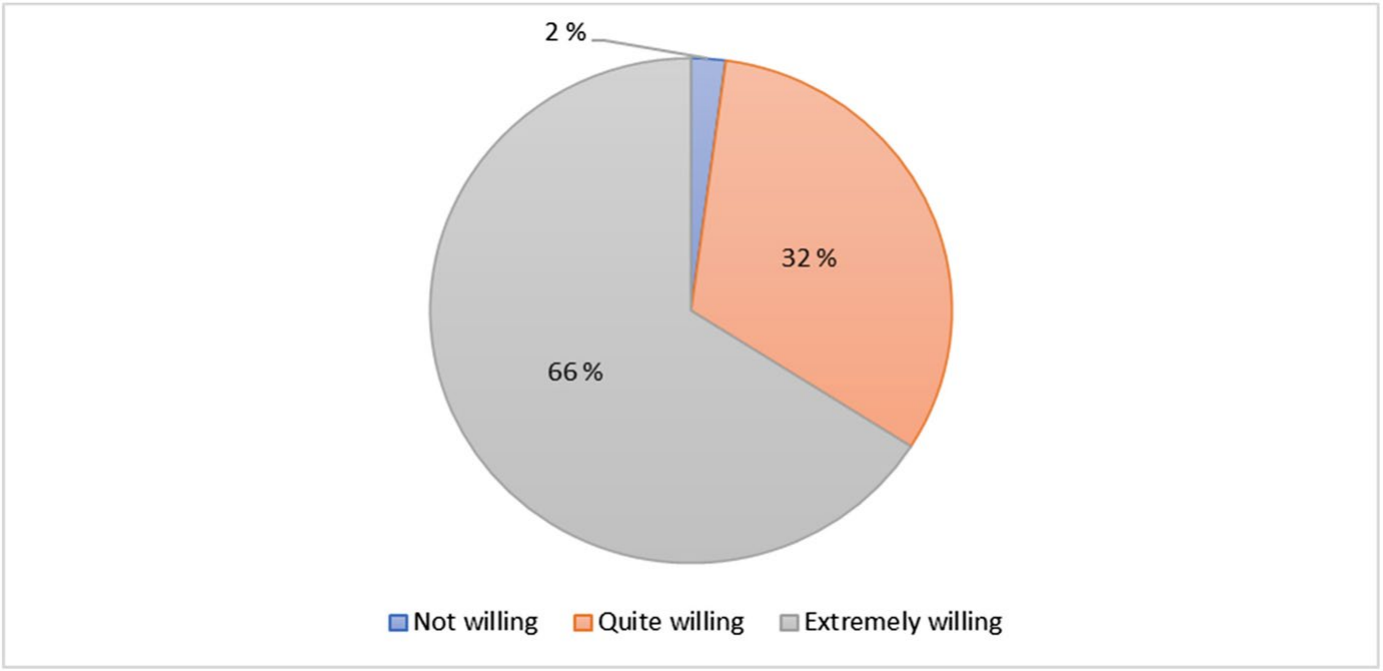
Recommendation of the HyFlex cyber training to other students’ distribution

Finally, results from Fig. 14 suggest that 66% (31) students are extremely willing to recommend the HyFlex cybersecurity training to other students. Another 32% (15) students are quite willing and only 1 (2%) student is not willing to recommend it to other students.

## Discussion and implications

This section presents a discussion of the results obtained in this study and their research and practical implications.

### Discussion

This study aimed to investigate the effectiveness of a HyFlex learning method in delivering cyber security training in a developing country. With the rapid evolution of cyber-attacks and the need for cyber security professionals with the required skills and competence to address these growing threats, HyFlex learning method provides an alternative mode of delivering for the much-needed cyber security education, especially in developing countries. Therefore, this study brings new perspectives by highlighting the implementation details of how the HyFlex delivery mode can be deployed in the context of a developing country, the benefits of such implementation, and the challenges that need to be considered during the implementation.

The findings from this study indicate that the implementation of a HyFlex learning method in a developing country requires special considerations. Even though several digital technological tools such as WhatsApp messaging tool, Zoom, Slack and Email, were employed for the implementation of the training, the setting of the study provided useful insights that could be used for implementing similar training in developing countries. For example, most of the participants that joined the training sessions remotely utilised mobile phones. This entails that the technology employed for the training needs to be optimised for mobile phones. Similarly, the course content and materials should be easily accessible using mobile phones to provide the students with the opportunity of actively engaging with the learning processes. There is also the issue of Internet connectivity, which most of the participants observed to have negatively impacted their ability to actively participate in the training sessions. To address this, it is imperative that the training session should be recorded to allow the participants to go over the video later.

Moreover, the results from this study reveal that the HyFlex learning method was able to accommodate participants with different levels of education and their life circumstances. It also provided access to a large number of participants and enabled them to decide the best way to join the training sessions and as such, gave them a sense of control over their learning. This is consistent with the results from Abdelmalak and Parra (2016) which observed that HyFlex course design was able to accommodate student needs and their life circumstances, increase student access to course content and instruction, and gave students a sense of control over their learning. Also, in line with the results from previous studies (Lakhal et al., 2014; Miller et al., 2013), the feedback obtained from the participants show that there was no significant difference in the outcome between the participants that joined remotely and those that participated through in-person meetings.

Interestingly, student engagement in the HyFlex learning method was examined in this study by considering the students opinion about the helpfulness of the coordinators and their perception about the support provided by the trainers both during the online and physical lectures. The results obtained indicate that most of the students were satisfied with the helpfulness of the coordinators and the support they provided, which demonstrates that the HyFlex delivery method promotes student engagement. These results are in line with the work from Heilporn and Lakhal (2021), which observed that the HyFlex learning method is a promising course modality for encouraging student engagement, especially in large-group courses.

Finally, another significant finding from this study relates to the student's overall perception of the HyFlex learning method. The results show that most of the students enjoyed the HyFlex pedagogical approach and are willing to recommend the HyFlex cyber security training to other students. However, the students were constrained in several ways by the available technology. For example, most of the students reported that the lack of Internet data or Internet connection and the unavailability of suitable technological tools negatively impacted their learning process during the training. This echoes the results from Binnewies and Wang (2019) which indicated that most students appreciated the HyFlex model of delivery but are constrained in some way by the available technology. Furthermore, it was interesting to notice that joining the cyber security training sessions remotely was the preferred mode of participation for most of the participants. This is an important result because it would help those implementing the HyFlex learning method in the planning and design of HyFlex course content in such a way as to engage participants joining remotely.

### Implications of the study

This research has several implications towards improving learning and teaching in higher education. The main contribution of this study focuses on examining the effectiveness of HyFlex in a cyber security course. The findings from this study provide evidence in the evolving research area of blended learning which offers a flexible student-centered teaching approach. The findings present an overview, benefits, and issues in HyFlex pedagogy faced in higher education. Overall, the findings from the survey indicates that students found the HyFlex course approach to be useful and beneficial to them as it improves their learning environment.

This study provides implications to lecturers suggesting that HyFlex is a useful method as it provides greater degree of flexibility to students. It also promotes the growth and advancement of digital course delivery in universities and colleges as it offers convenience and access as compared to traditional face-to-face teaching delivery. The factors that influence students' adoption of HyFlex in a developing country context are also presented. Furthermore, HyFlex offers an aspect that extends the usefulness of digital based learning and interactive course delivery. HyFlex aids to improve learners’ engagement in achieving an engaging digital and physical learning environment. In addition, it assists in stimulating learners’ interaction to actualize a more community, and collaborative-based learning. Evidently, HyFlex can be employed by teachers to foster different course delivery modalities in balancing flexibility in distance courses delivery and in-person courses amidst the COVID-19 pandemic.

## Conclusion

This study investigated the effectiveness of a HyFlex cyber security training in the context of a developing country. The findings indicate that there are special considerations in terms of the available technological tools, Internet connectivity and electric power related issues, during the implementation of a HyFlex cyber training from the context of a developing country. Findings from this study also suggest that the HyFlex pedagogical method is able to accommodate a large number of participants with different levels of education and their life circumstances and there is no significant difference in the outcome between the participants that joined remotely and those that participated through in-person meetings.

More importantly, this study examines two research question which provided practical evidence on how HyFlex be implemented to utilize digital technologies in a cyber security training in a developing country context as seen in the methodology section of this paper. Also, findings from this study presented the factors that influence students’ perception on use of digital synchronous and asynchronous learning in a cyber security training grounded on results from the survey questionnaires as presented in results section of this paper. Moreover, the results from this study reveal that students enjoyed the HyFlex delivery method as it promotes student engagement, and they are willing to recommend it to other students. In addition, this study provides implications to educationalists towards improving learning and teaching in higher education.

Also, this study has few limitations. One of the limitations of this study is that the lecturer's perspective was not well explored in this current study. The study was more aligned to the students' perception towards the adoption of HyFlex pedagogy to support cyber security training in a developing country context. Secondly, this study employed a low dataset from only 47 usable samples. But the validity of the results is credible since we had more than 40% response rate. Finally, this study did not employ Quasi-experimental study as carried out by Zehler et al. (2021) where the authors deployed a Hyflex simulation towards creative method to unprecedented circumstances. As such this study did not provide more in-depth experimental results in a quantitative manner for pre-test and post-test to further test the HyFlex model against previous pedagogical model in the literature.

Future works will include exploring the perception of academicians towards the adoption of HyFlex pedagogy. Moreover, more data will be collected from students to improve this research study. Lastly, experimental results in a quantitative manner for pre-test and post-test will be provided to offer further evidence on student’s perception towards HyFlex pedagogical approach.
